# Complete chloroplast genome of *Engelhardtia fenzlii* (Juglandaceae)

**DOI:** 10.1080/23802359.2020.1863871

**Published:** 2021-01-27

**Authors:** Mu Liu, Jinsen Lu, Yuan Li, Lvshui Zhang

**Affiliations:** College of Landscape Architecture and Arts, Jiangxi Agricultural University, Nanchang, China

**Keywords:** Chloroplast genome, *Engelhardtia fenzlii*, phylogeny

## Abstract

In this study, we successfully assembled and analyzed the chloroplast genome of *Engelhardtia fenzlii*. The chloroplast genome of *E. fenzlii* was very similar to those of other Juglandaceae species. The *E. fenzlii* chloroplast genome is 161,713 bp in length and displays the typical quadripartite structure, which consists of a pair of IR regions (26,016 bp) separated by an LSC region (90,478 bp) and an SSC region (19,203 bp). The chloroplast genome of *E. fenzlii* contained a total of 112 unique genes, including 78 protein-coding genes, 30 tRNAs, and 4 rRNAs. Phylogenetic analysis based on the complete chloroplast genomes showed that *Engelhardtia* formed a monophyletic clade and *E. fenzlii* was sister to *E. roxburghiana.*

*Engelhardia* Leschen. ex Blume is a genus of deciduous or evergreen trees in the walnut family (Juglandaceae) and used to be traditional medicines and health tea. *Engelhardtia fenzlii* is an evergreen tree species that grows at an altitude of 400 to 1000 m in south China, and traditionally used in papermaking. The chloroplast genomes are widely used in research on comparative genomics, plant system development, and phylogeny at different taxonomic ranks (Dong et al. 2018, 2020). In this study, we sequenced the complete chloroplast genome of *E. fenzlii* using Illumina Hiseq X ten platform. Our objective was to provide information for the systematic evolution studies of Juglandaceae, with special interest in the positioning of *E. fenzlii* in plant systematics and evolution.

Fresh and young leaves of *E. fenzlii* were collected from Gannan Arboretum, Jiangxi, China (25°51′10″∼114°22′25″) for DNA extraction. Voucher specimen was deposited at the herbarium of Jiangxi Agricultural University with the specimen voucher number of LM850486. Total genomic DNA was isolated using the Mag-MK Plant Genomic DNA extraction kit (Sangon Biotech, Shanghai, China) for constructing a shotgun library and sequencing on an Illumina Hiseq X ten platform. Low-quality reads and adapters were filtered from the raw data by using Trimmomatic (Bolger et al. 2014). The chloroplast genome was assembled with GetOrganelle (Jin et al. 2019). Plastomes were annotated with Plann (Huang and Cronk 2015) using *Juglans regia* (Dong et al. 2017) as reference. The annotated chloroplast genome has been deposited into GenBank with the accession number of MT991009.

The *E. fenzlii* chloroplast genome structure was similar to those of other Juglandaceae species (Dong et al. 2017). The whole chloroplast genome of *E. fenzlii* is 161,713 bp in length and displays the typical quadripartite structure, which consists of a pair of IR regions (26,016 bp) separated by an LSC region (90,478 bp) and an SSC region (19,203 bp). The *E. fenzlii* chloroplast genome possessed 112 unique genes, including 78 protein-coding, 30 tRNA, and 4 rRNA genes. GC content is 35.9%. Introns occur in 19 genes, with 17 of them having one intron while two genes (*clpP* and *ycf3*) have two introns. In *rps12*, a trans-splicing event was observed, with the 5′ end located in the LSC region and the duplicated 3′ end in the IR region. The *trnK-UUU* gene harbors the largest intron, which contains the *matK* gene.

To examine the phylogenetic position of *E. fenzlii*, Maximum Likelihood (ML) method of phylogenetic analysis was performed based on whole chloroplast genomes from 26 Juglandaceae species. The chloroplast genome sequences were aligned using MAFFT v7 (Katoh and Standley 2013). Ambiguous alignment regions were trimmed by Gblocks 0.91 b (Castresana 2002). The ML analyses were conducted using RAxML 8.0 (Stamatakis 2014). For ML analysis, the best-fit model, general time reversible (GTR)+G was used as suggested with 1,000 bootstrap replicates.

The monophyly of Juglandaceae was strongly supported based on the available whole complete chloroplast genome dataset. All the genera except *Annamocarya* in Juglandaceae were fully supported as monophyletic (bs = 100). The tribe Juglandeae was well supported, consisting of *Cyclocarya*, *Juglans* and *Pterocarya* and was sister to *Platycarya. E. fenzlii* was sister to *E. roxburghiana* and formed a monophyletic clade. In summary, this study will be helpful for further research on the molecular evolution and speciation of *Engelhardtia* (Figure 1).

**Figure 1.**
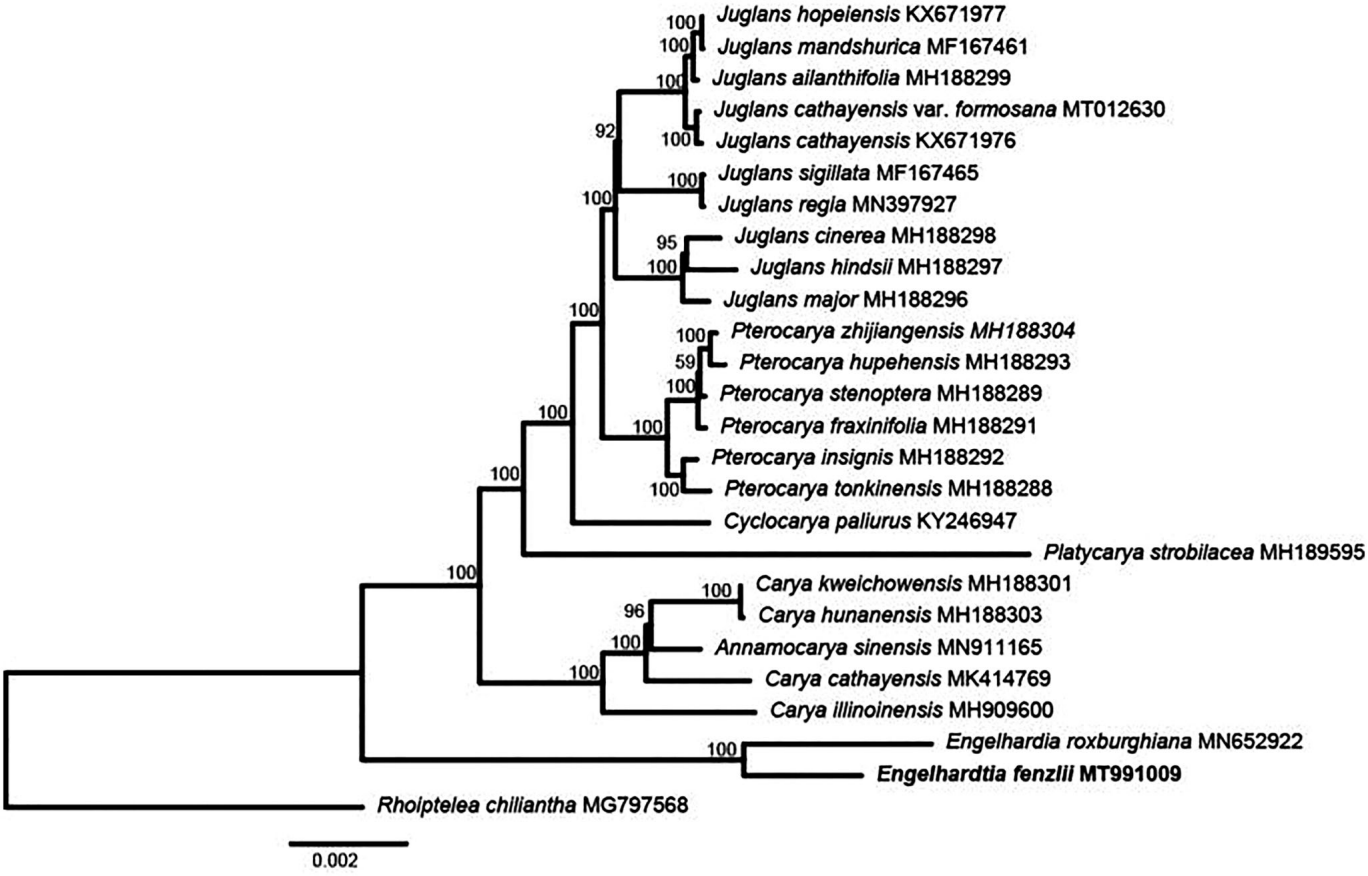
Phylogenomic relationships of Juglandaceae based on the complete chloroplast genome sequences. Maximum likelihood (ML) topology was shown with ML bootstrap support value presented at each node. Bootstrap support values >50% are given at the nodes.

## Data Availability

The chloroplast genome sequence of the *E. fenzlii* was submitted to GenBank of NCBI. The accession number from GenBank is MT991009 (https://www.ncbi.nlm.nih.gov/nuccore/MT991009). The raw data has been deposited in SRA under accession number SRR12628799 (https://www.ncbi.nlm.nih.gov/sra/?term=SRR12628799).

## References

[CIT0001] Bolger AM, Lohse M, Usadel B. 2014. Trimmomatic: a flexible trimmer for Illumina sequence data. Bioinformatics. 30(15):2114–2120. 24695404 10.1093/bioinformatics/btu170 PMC4103590

[CIT0002] Castresana J. 2002. GBLOCKS: selection of conserved blocks from multiple alignments for their use in phylogenetic analysis. Mol Biol Evol. 17(4):540–552. 10.1093/oxfordjournals.molbev.a026334 10742046

[CIT0003] Dong W, Xu C, Li W, Xie X, Lu Y, Liu Y, Jin X, Suo Z. 2017. Phylogenetic resolution in *Juglans* based on complete chloroplast genomes and nuclear DNA sequences. Front Plant Sci. 8:1148. 28713409 10.3389/fpls.2017.01148 PMC5492656

[CIT0004] Dong W, Xu C, Wen J, Zhou S. 2020. Evolutionary directions of single nucleotide substitutions and structural mutations in the chloroplast genomes of the family Calycanthaceae. BMC Evol Biol. 20(1):96. 32736519 10.1186/s12862-020-01661-0 PMC7393888

[CIT0005] Dong W, Xu C, Wu P, Cheng T, Yu J, Zhou S, Hong D-Y. 2018. Resolving the systematic positions of enigmatic taxa: manipulating the chloroplast genome data of Saxifragales. Mol Phylogenet Evol. 126:321–330. 29702217 10.1016/j.ympev.2018.04.033

[CIT0006] Huang DI, Cronk QCB. 2015. Plann: a command-line application for annotating plastome sequences. Appl Plant Sci. 3(8):1500026. 10.3732/apps.1500026 PMC4542940 26312193

[CIT0007] Jin J-J, Yu W-B, Yang J-B, et al. 2019. GetOrganelle: a fast and versatile toolkit for accurate de novo assembly of organelle genomes. bioRxiv, 256479. 10.1186/s13059-020-02154-5 PMC7488116 32912315

[CIT0008] Katoh K, Standley DM. 2013. MAFFT multiple sequence alignment software version 7: improvements in performance and usability. Mol Biol Evol. 30(4):772–780. 23329690 10.1093/molbev/mst010 PMC3603318

[CIT0009] Stamatakis A. 2014. RAxML version 8: a tool for phylogenetic analysis and post-analysis of large phylogenies. Bioinformatics. 30(9):1312–1313. 24451623 10.1093/bioinformatics/btu033 PMC3998144

